# HTLV-1 Extracellular Vesicles Promote Cell-to-Cell Contact

**DOI:** 10.3389/fmicb.2019.02147

**Published:** 2019-09-18

**Authors:** Daniel O. Pinto, Catherine DeMarino, Michelle L. Pleet, Maria Cowen, Heather Branscome, Sarah Al Sharif, Jennifer Jones, Helene Dutartre, Benjamin Lepene, Lance A. Liotta, Renaud Mahieux, Fatah Kashanchi

**Affiliations:** ^1^Laboratory of Molecular Virology, School of Systems Biology, George Mason University, Manassas, VA, United States; ^2^Vaccine Branch, National Cancer Institute, National Institutes of Health, Bethesda, MD, United States; ^3^International Center for Research in Infectiology, Retroviral Oncogenesis Laboratory, INSERM U1111-Université Claude Bernard Lyon 1, CNRS, UMR5308, Ecole Normale Supérieure de Lyon, Université de Lyon, Fondation pour la Recherche Médicale, Labex Ecofect, Lyon, France; ^4^Ceres Nanosciences, Inc., Manassas, VA, United States; ^5^Center for Applied Proteomics and Molecular Medicine, George Mason University, Manassas, VA, United States

**Keywords:** HTLV-1, extracellular vesicle, viral spread, tax, cell-to-cell contact, infection, RNA, DNA

## Abstract

Human T-cell leukemia virus-1 (HTLV-1) is a neglected and incurable retrovirus estimated to infect 5 to 10 million worldwide. Specific indigenous Australian populations report infection rates of more than 40%, suggesting a potential evolution of the virus with global implications. HTLV-1 causes adult T-cell leukemia/lymphoma (ATLL), and a neurological disease named HTLV-1 associated myelopathy/tropical spastic paraparesis (HAM/TSP). Even though HTLV-1 transmission primarily occurs from cell-to-cell, there is still a gap of knowledge regarding the mechanisms of viral spread and disease progression. We have recently shown that Extracellular Vesicles (EVs) ubiquitously produced by cells may be used by HTLV-1 to transport viral proteins and RNA, and elicit adverse effects on recipient uninfected cells. The viral proteins Tax and HBZ are involved in disease progression and impairment of autophagy in infected cells. Here, we show that activation of HTLV-1 via ionizing radiation (IR) causes a significant increase of intracellular Tax, but not EV-associated Tax. Also, lower density EVs from HTLV-1-infected cells, separated by an Iodixanol density gradient, are positive for gp61+++/Tax+++/HBZ+ proteins (HTLV-1 EVs). We found that HTLV-1 EVs are not infectious when tested in multiple cell lines. However, these EVs promote cell-to-cell contact of uninfected cells, a phenotype which was enhanced with IR, potentially promoting viral spread. We treated humanized NOG mice with HTLV-1 EVs prior to infection and observed an increase in viral RNA synthesis in mice compared to control (EVs from uninfected cells). Proviral DNA levels were also quantified in blood, lung, spleen, liver, and brain post-treatment with HTLV-1 EVs, and we observed a consistent increase in viral DNA levels across all tissues, especially the brain. Finally, we show direct implications of EVs in viral spread and disease progression and suggest a two-step model of infection including the release of EVs from donor cells and recruitment of recipient cells as well as an increase in recipient cell-to-cell contact promoting viral spread.

## Introduction

Human T-cell leukemia virus-1 (HTLV-1) is a neglected blood-borne pathogen that indiscriminately infects people of all ages around the world (Gallo, 2011; Gallo et al., 2016; Tagaya and Gallo, 2017; Martin et al., 2018). Approximately 5–10 million people worldwide carry HTLV-1, although this estimate does not include epidemiological data from highly populated regions, such as China, India, Maghreb, and East Africa, and very limited epidemiological data from Africa where the virus is endemic. Therefore actual numbers may be closer to 20 million (Kondo et al., 1989; Koga et al., 2010; Gessain and Cassar, 2012; Einsiedel et al., 2016). More recently, reports of high HTLV-1 infection rates, of 40% and above, in remote Australian populations are cause of global concern (Einsiedel et al., 2016, 2018). HTLV-1-infected individuals remain asymptomatic and possibly unaware of their infection status (Hinuma et al., 1981; Gonçalves et al., 2010; Gessain and Cassar, 2012), which is especially concerning since a significant route of transmission occurs from mother to child via breastfeeding (Fujino and Nagata, 2000; Einsiedel et al., 2016). Moreover, it is likely that, due to a lack of universal screening recommendations, more people globally are at risk of infection via sexual contact, blood transfusions and organ transplants (Gallo et al., 2016; de Morais et al., 2017; Caswell et al., 2019).

Human T-cell leukemia virus-1 is associated with Adult T-cell leukemia/lymphoma [ATLL; 3–5% (Hinuma et al., 1981; Poiesz et al., 1981; Aoki et al., 1984; Kondo et al., 1989; Hirai et al., 1992; Iwanaga et al., 2012; Coffin, 2015; Tagaya and Gallo, 2017)] and HTLV-1 associated myelopathy/tropical spastic paraparesis [HAM/TSP; 0.25–3.8% (Gessain et al., 1985; Kaplan et al., 1990; Bangham et al., 2015; Naito et al., 2018)]. Recently, another HTLV-1-associated disease, bronchiectasis, has been linked to high mortality rates (28%) in HTLV-1 seropositive patients (Einsiedel et al., 2018). Depending on the country, current ATLL treatments may include a combination of interferon (IFN) and Zidovudine (AZT), allogeneic stem cell transplantation, or mogamulizumab, a humanized anti-CCR4 monoclonal antibody (Gill et al., 1995; Hermine et al., 2002; Tsukasaki et al., 2009; Subramaniam et al., 2012), however, these treatments are ineffective against some ATLL forms, and in cases of relapse (Phillips et al., 2016). Due to chronic stage of HTLV-1 infection, disease progression may be stalled for several years in an asymptomatic state (latent), with increasing risk over time of developing ATLL (Iwanaga et al., 2010) or HAM/TSP, and of transmitting HTLV-1.

In contrast to human immunodeficiency virus type-1 (HIV-1), HTLV-1 transmission occurs primarily via cell-to-cell contact and formation of a virological synapse, viral biofilm, cellular conduit, or tunneling nanotube (Igakura et al., 2003; Majorovits et al., 2008; Nejmeddine et al., 2009; Pique and Jones, 2012; Alais et al., 2015; Wang et al., 2017; Omsland et al., 2018). Characterization of the proteins involved in viral transmission suggests participation of adhesion proteins (i.e., ICAM-1 and LFA-1), viral proteins [i.e., p12, p8, gp61 (precursor of gp46 envelope), and Tax], and cellular proteins (i.e., Hsc70, CD43, and CD45) (Igakura et al., 2003; Nejmeddine et al., 2009; Van Prooyen et al., 2010b; Mazurov et al., 2012; Pique and Jones, 2012; Pise-Masison et al., 2014; Dutartre et al., 2016; Gross and Thoma-Kress, 2016; Gross et al., 2016; Futsch et al., 2017; Omsland et al., 2018). Although cell surface and viral proteins have been identified as players in cell-to-cell adhesion, there is still a gap of knowledge regarding the mechanisms by which HTLV-1 may increase viral spread. We propose that the small membrane-bound structures, known as Extracellular Vesicles (EVs), contribute to recipient cell recruitment and increased HTLV-1 viral spread.

Extracellular Vesicles have gained considerable attention due to their increasingly apparent role in cell-to-cell communication and in mediating disease (Gould et al., 2003; Keller et al., 2006; Fleming et al., 2014; Jaworski et al., 2014a; Sampey et al., 2014; Vader et al., 2014; Ahsan et al., 2016; Anderson et al., 2016; Pleet et al., 2017, 2018a,b; Barclay et al., 2017b; Anderson M. et al., 2018; DeMarino et al., 2018). EVs encompass a wide range of sizes based on their biogenesis pathway, such as exosomes (50–100 nm) and microvesicles (50–10,000 nm) (Keller et al., 2006; Raposo and Stoorvogel, 2013; Fleming et al., 2014; Kastelowitz and Yin, 2014; Sampey et al., 2014; Vader et al., 2014; van Dongen et al., 2016; van Niel et al., 2018). The detection of viral proteins in EVs secreted from infected cells denotes their potential to mediate disease. For instance, we have previously shown that the HTLV-1 protein Tax, implicated in driving oncogenesis and subsequent development of ATLL (Baratella et al., 2017; Billman et al., 2017; Tagaya and Gallo, 2017; Mahgoub et al., 2018), may be found in EVs from infected cells (Jaworski et al., 2014a). Also, we recently found that HAM/TSP patient cerebral spinal fluid (CSF) and peripheral blood mononuclear cells (PBMCs) consistently express Tax associated with EVs that may trigger inflammatory responses known to cause neurodegeneration (Anderson M.R. et al., 2018). However, it is still unclear which type of EV and associated cargo may participate in HTLV-1 pathogenesis, as well as the extent of the cellular targets (i.e., neuronal, myeloid, and T-cells) that may be involved in this pathogen-EV-host interaction.

Our lab has previously shown that low levels of ionizing radiation (IR) can be used to activate latent viral reservoirs *in vitro* and *in vivo* across multiple tissues (blood, liver, lung, brain, and spleen) (Iordanskiy et al., 2015; Iordanskiy and Kashanchi, 2016). IR is also used as a tool to block cell cycle progression of HTLV-1-infected cells prior to administration in animal models of HTLV-1 infection (Tezuka et al., 2014, 2018). In this manuscript, we initially used IR as a probe to study HTLV-1 in a transcriptionally active setting, as to better resemble patients expressing higher levels of viral transcripts. We further explored the potential uses of IR in modulating EV release, as well as viral activation. Specific EV types derived from infected cells in distinct transcriptional states may potentially elicit varied effects on neighboring cells, such as activating uninfected T-cells or promoting viral spread. Understanding the mechanistic differences between latent and transcriptionally active HTLV-1 may allow for the development of clinical tools in the early detection of disease (i.e., EV/viral biomarkers) important for ATLL or HAM/TSP.

Here, we have attempted to address whether treatments such as IR affect EV release and cargo packaging (i.e., gp61^+++^/Tax^+++^/HBZ^+^; referred to as HTLV-1 EVs). We characterized the cargo of HTLV-1 EVs separated by a novel technique to isolate virus away from EVs. Additionally, we tested the functional role of EVs in promoting cell-to-cell contact and subsequent viral spread and identified CD45 and ICAM-1 as possible players in EV-mediated cell-to-cell contact. Finally, we examined the *in vivo* functional roles of HTLV-1 EVs in promoting spread and proviral integration. Collectively, we propose a novel two-step model of HTLV-1 infection, which involves EV-mediated priming of uninfected recipient cells and increased cell-to-cell contact resulting in an enhanced viral spread.

## Results

### Viral Activation via IR Increases Intracellular Tax and EV Release

Our previous studies have shown that Tax protein may be encapsulated in EVs isolated from HTLV-1-infected cells (Jaworski et al., 2014a). Additionally, our more recent data have shown that EV-associated Tax can be isolated from HAM/TSP patient PBMCs and CSF samples (Anderson M.R. et al., 2018). These data demonstrate the potential clinical relevance and functional roles of EVs in HTLV-1 infection. We sought to elucidate the potential functional roles of EVs in HTLV-1 infection, particularly concerning viral spread. We wanted to understand the fundamental differences in Tax expression and EV release between latent and activated viral settings using ionizing radiation (IR), which can be used to activate virus (Iordanskiy et al., 2015). HTLV-1-infected HUT102 cells were treated with IR (10 Gy) and then incubated for 5 days to allow for maximal EV release, as described previously for HTLV-1 and other viruses (Narayanan et al., 2013; Jaworski et al., 2014a, b; Sampey et al., 2016; Barclay et al., 2017b; Anderson M.R. et al., 2018).

**FIGURE 1:**
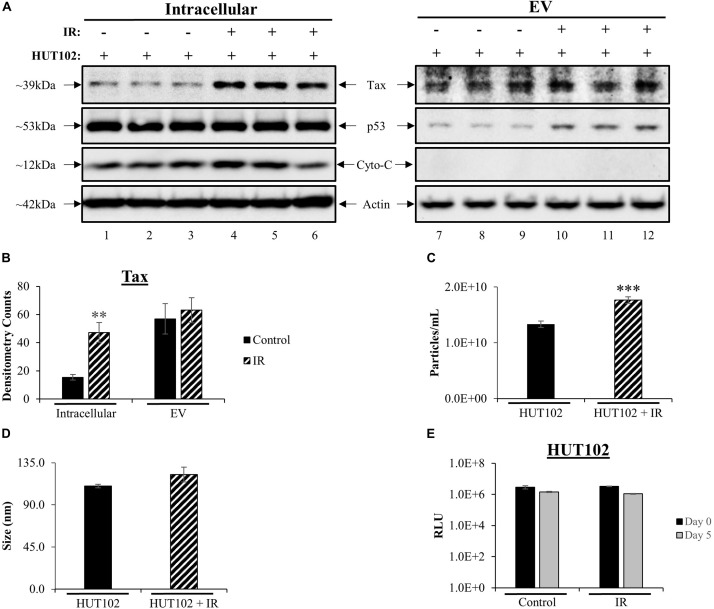
Viral Activation with IR Increases Intracellular Tax and EV Release. HUT102 cells (1 × 10^6^ cells/mL) from three distinct cultures were treated with IR (10 Gy) and cultured for 5 days in EV-depleted media. **(A)** From 1 mL of culture, cell pellets were lysed, and EVs captured and enriched using Nanotrap (NT) particles NT080/NT082. WB analysis was performed on cell lysates (lanes 1–6) and EVs (lanes 7–12) for detection of Tax, p53 (IR control), Cytochrome c (negative control for EV) and Actin protein expression. **(B)** Densitometry analysis was used for each Tax band and normalized to its corresponding Actin band. Biological triplicates were averaged together (lanes 1–3, 4–6, 7–9, and 10–12) and a two-tailed student *t*-test used to evaluate statistical significance between control (no treatment) and IR treatment. **(C,D)** ZetaView analysis of supernatant material derived from 1 × 10^6^ HUT102 cells/mL (±IR; 10 Gy) was analyzed to determine EV size and concentration from three technical replicates. ZetaView analysis of EVs from HUT102 cells was evaluated in multiple independent experiments (*n* > 3) and the trends were replicated consistently. **(E)** Cell viability was evaluated for HUT102 cells at 1 × 10^6^ cells/mL for control and IR conditions after 5 days. A two-tailed student *t*-test was performed to evaluate statistical significance with ^∗∗∗^*p*-value ≤ 0.001 indicating the highest statistical significance, followed by ^∗∗^*p*-value ≤ 0.01.

Western blot (WB) analysis was used to assess intracellular and EV-associated Tax protein expression levels related to viral activation. Intracellular Tax levels increased after treatment with IR (Figure 1A; lanes 1–3 vs. 4–6), while no increase in EV-associated Tax levels were observed (Figure 1A; lanes 7, 8 vs. 10–12). The p53 protein is typically used as a marker for double-stranded DNA damage induced by irradiation. HTLV-1 infected cells express high levels of inactive p53 (Yamato et al., 1993; Mori et al., 1997; Mulloy et al., 1998; Pise-Masison et al., 1998, 2000; de la Fuente et al., 2000; Mahieux et al., 2000; Grassmann et al., 2005; Pise-Masison and Brady, 2005). As expected, intracellular p53 levels were unchanged post-IR treatment (Figure 1A; compare lanes 1–6) however, p53 levels in EVs were increased after IR (compare lanes 7–12). Cytochrome c protein levels were also evaluated as a negative control for EVs (Figure 1A; lanes 7–12; as previously described in Jaworski et al., 2014a). Signal quantification was performed and confirmed a significant increase only in intracellular Tax (Figure 1B). These data potentially suggest that the mechanisms driving Tax protein packaging into EVs are either not altered by viral activation or are close to their maximal capacity. Alternatively, IR and an excess of Tax may also activate autophagy that degrades excess Tax that may have otherwise been secreted in EVs.

We next asked whether IR had any effects on EVs release from HTLV-1-infected cells. Nanotracking analysis (NTA; ZetaView) was used to quantitate EVs isolated from HTLV-1-infected cells (Figure 1C). This revealed a significant increase (^∗∗∗^*p* ≤ 0.001) for HUT102 cells treated with IR. The overall diameter of EVs did not change between control and IR lanes (Figure 1D). Finally, to ensure that culture settings and IR treatment were not deleterious to HUT102 cells, cell viability was measured at day 0 and day 5 for control and IR treated cells, revealing no significant change (Figure 1E). Uninfected T-cells (CEM) were also assayed under the same culture conditions revealing no significant change in viability (data not shown; Supplementary Figure S1). Altogether, these data suggest that IR may cause an increase in EV release from HTLV-1-infected cells without significantly altering EV biogenesis pathways, as per the unchanged EV diameter measured before and after treatment.

### IR Activates HTLV-1 Transcription Without Increasing Viral RNA Levels in EVs

**FIGURE 2:**
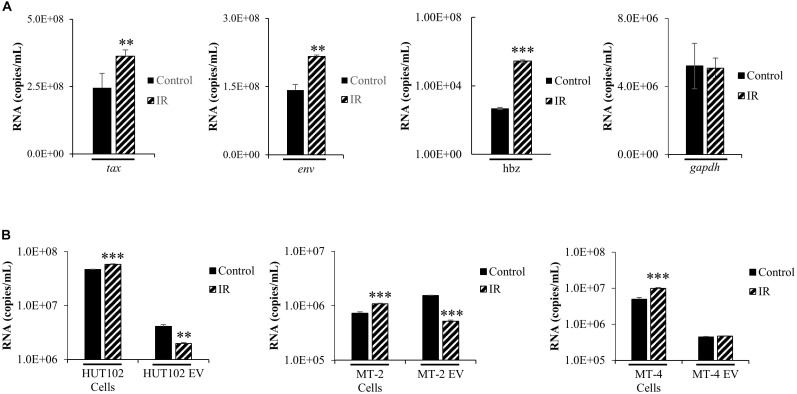
HTLV-1 Activation Increases Intracellular Viral RNA Transcription, but not RNA packaging into EVs. HUT102 cells (1 × 10^6^ cells/mL) were treated (±IR; 10 Gy) and cultured as described previously. **(A)** RNA was isolated from a 1 mL culture of HUT102 cells for analysis by RT-qPCR of *tax*, *env*, *hbz, and gapdh.* At least three independent experiments, each in technical triplicate, were performed and observed consistent reproducibility. **(B)** Similarly, 1 mL cultures of HUT102, MT-2, and MT-4 cells were cultured and EVs nanotrapped (NT080/082) and used for RT-qPCR analysis of *env* RNA. RT-qPCRs were performed in technical triplicate. A two-tailed student *t*-test was used to evaluate statistical significance with ^∗∗∗^*p*-values ≤ 0.001, ^∗∗^*p*-values ≤ 0.01, and *^∗^p*-values ≤ 0.05 indicating the level of significance.

The functional roles of EVs in transport of viral proteins and RNA is a topic of significant importance to the understanding of overall viral pathogenesis, especially since EVs have been shown to modulate immune responses (Muller et al., 2016; Pleet et al., 2016; Sampey et al., 2016; Anderson M.R. et al., 2018) and package viral cargo that may regulate gene expression (Ung et al., 2014; Barclay et al., 2017b). For instance, we have recently observed that in chronic HIV-1 infection, EVs from uninfected cells may activate viral transcription (Barclay et al., 2017b). In the case of HTLV-1, we have previously shown that viral RNA may be packaged into EVs released from infected cells (Jaworski et al., 2014a). These observations highlight the importance of understanding the cargo associated with EVs and its potential effects on recipient cells. We hypothesized that IR would increase HTLV-1 RNA transcripts intracellularly and that this increase would also result in increased incorporation of HTLV-1 RNA into EVs. Intracellular HTLV-1 *env*, *tax*, and *hbz* transcripts were quantified by RT-qPCR in HUT102 cells treated with IR (Figure 2A). Viral activation via IR specifically increased intracellular levels of *tax* (2.346 × 10^8^ to 3.63 × 10^8^ copies/mL; *p*-value ≤ 0.05), *env* (1.42 × 10^8^ to 2.16 × 10^8^ copies/mL; *p*-value ≤ 0.01), and *hbz* RNA (4.78 × 10^2^ to 2.80 × 10^5^ copies/mL; *p*-value ≤ 0.001) (Figure 2A). However, *gapdh* cellular housekeeping gene remained unaffected by IR treatment. Overall, these data suggest that IR selectively promotes HTLV-1 transcription at both the 5′ and 3′ long terminal repeat (LTR).

To validate observations from Figure 2A, an experiment was performed using MT-2 and MT-4 HTLV-1-infected cell lines, with and without IR treatment (Figure 2B). RT-qPCR was performed specifically for *env* RNA since it generates the important precursor envelope protein gp61, and proteolytic byproducts gp46 and gp21. HTLV-1 envelope protein may have crucial roles in promoting cell-to-cell contact; therefore, it is important to study the effects of viral activation (i.e., IR) on *env* RNA transcription and packaging into EVs. RT-qPCR data revealed a statistically significant increase (*p*-value ≤ 0.001) in intracellular *env* in all three IR treated cell lines, HUT102 (4.67 × 10^7^ to 5.82 × 10^7^ copies/mL), MT-2 (7.28 × 10^5^ to 1.08 × 10^6^ copies/mL), and MT-4 (4.96 × 10^6^ to 9.88 × 10^6^ copies/mL; Figure 2B). However, we found a decrease in *env* RNA in EVs from HUT102 (4.09 × 10^6^ to 1.98 × 10^6^ copies/mL; *p*-value ≤ 0.01) and MT-2 (1.53 × 10^6^ to 5.18 × 10^5^ copies/mL; *p*-value ≤ 0.001), but not from MT-4 (4.42 × 10^5^ to 4.70 × 10^5^ copies/mL) EVs (Figure 2B). These data suggest that while IR causes an increase in overall intracellular *env* RNA across all three HTLV-1-infected cell lines, it does not increase its packaging into EVs.

### HTLV-1-Infected Cells Secrete EVs Containing Differentially Packaged Viral Proteins and RNA

**FIGURE 3:**
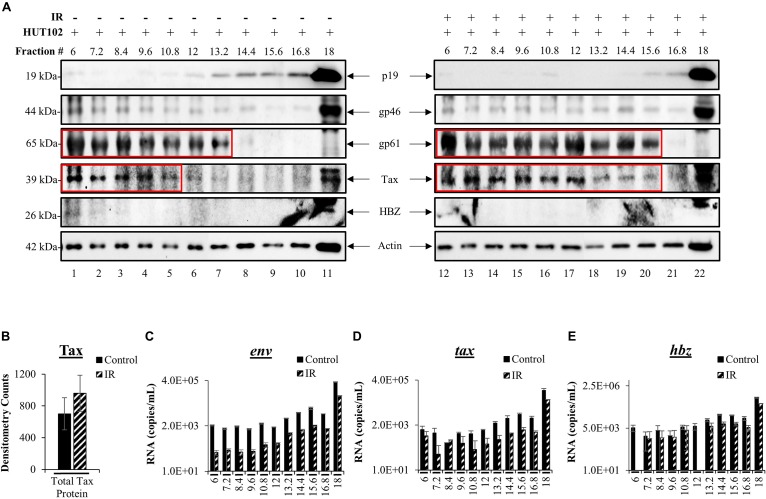
HTLV-1 proteins are differentially packaged after Viral Activation. HUT102 cells were treated with IR (10 Gy) and cultured in Exo-Free media for 5 days. Supernatants were collected from control and IR treated cells for precipitation by ExoMAX and separation into 11 fractions by OptiPrep/ultracentrifugation. Each resulting fraction was concentrated with NT080/082/086 particle mixture overnight. **(A)** WB analysis was conducted for each fraction from control (lanes 1–11) and IR treated cells (lanes 12–22), and probed for p19, gp61, gp46, HBZ, Actin. Each figure represents two blots with identical exposure combined into one for control and one for IR. **(B)** Densitometry analysis was performed on Tax protein bands from three independent Iodixanol gradients, averaged, and normalized to Actin. EV RNA was analyzed by RT-qPCR for **(C)** *env* RNA, **(D)** *tax* RNA, **(E)** and *hbz* RNA. Error bars represent one standard deviation above and below the averaged RNA copies/mL of technical triplicates.

We previously developed a novel EV isolation and separation method using density gradients, which allow isolation of EVs away from viruses and into distinct EV groups, large protein complexes, and free protein fraction (DeMarino et al., 2018, 2019). We reasoned that the differences in EV density are potentially due to a combination of EV cargo/packaging and size. The isolation method consists of the addition of ExoMax reagent for the precipitation of EVs, followed by the use of an Iodixanol gradient for the separation of distinct EV populations away from the potential virus. EVs from HUT102 cells (±IR) were separated into 11 fractions and then enriched with Nanotrap particles (NT080/082/086) for downstream detection of HTLV-1 matrix (p19), envelope (gp61 and gp46), Tax, HBZ, and Actin (control) proteins. WB analysis showed the presence of p19 in fractions 13.2 to 18 (Figure 3A; lanes 7–11), while cells treated with IR yielded EVs that primarily displayed p19 in fraction 18 (Figure 3A; lane 22). This finding is in line with previous observations in the context of HIV-1 infection which suggest that fraction 18 may contain a mixture of EVs and potentially virus, or at least an abundance of viral components in autophagosomes (DeMarino et al., 2018). Interestingly, mature envelope protein, gp46, was almost exclusively present in fraction 18, unaffected by IR (Figure 3A; lanes 11 and 22). Low levels of gp46 were also detected in the left side of the gradient mainly at fraction 6. Not surprisingly, colocalization of gp46 with p19 (on the right side of the gradient; fraction 18) suggests the presence of a potential viral particle, whereas the presence of gp46 in fraction 6 may suggest free gp46. On the other hand, a strong signal for the unprocessed form of HTLV-1 envelope protein (gp61) was detected in lower density fractions 6 to 13.2 (lanes 1–7) and in IR treated fractions 6 to 15.6 (lanes 12–20), while absent in high-density fractions 16.8 and 18 (lanes 10, 11 and 21, 22). This data provides further confirmation that processed proteins and virus may colocalize in the high-density fraction 18 (lanes 11–22), and unprocessed proteins in low-density EV fractions. Next, Tax was detected in lower density fractions 6 to 10.8 (lanes 1–5). However, upon IR treatment, a shift in Tax packaging was observed into the higher density fractions (12 to 15.6; lanes 17–20). The signal for Tax was strong in fraction 18 (lane 11) and more so in IR treated fraction 18 (lane 22). These findings suggest that upon IR treatment Tax is potentially packaged into higher density EVs, potentially due to the increased transcription of *tax* mRNA as previously observed in Figure 2A. Finally, we observed low levels of anti-sense protein, HBZ, in fractions 6 and 18 without IR treatment, while IR treated fractions contained almost no HBZ protein.

To more closely inspect the overall levels of Tax in EVs after viral activation, we performed densitometry analysis of gradient separated Tax bands from three distinct biological replicates and found that IR caused a non-significant increase of the total Tax protein levels (Figure 3B). Next, RT-qPCR analysis of each fraction was performed to characterize HTLV-1 *env, tax, hbz* RNA levels in EVs. In Figure 3C, fractions 6 to 12 contained very similar quantities of *env* RNA, with fraction 6 quantifying an average of 2.11 × 10^3^ copies (control) and 9.00 × 10 copies (IR) of total *env* RNA. Higher *env* RNA levels were detected as the density of the fractions increased, with the most abundant quantities of RNA in fraction 18 [3.4 × 10^5^ (control) and 6.7 × 10^4^ copies (IR)] (Figure 3C). IR treatment resulted in a decrease in *env* RNA levels in every fraction (Figure 3C, fractions 6 to 18), potentially due to alterations in packaging. However, it is important to note that *env* RNA levels could be the result of amplification of both *env* mRNA and genomic RNA. Similar trends were observed for both *tax* (Figure 3D) and *hbz* (Figure 3E). RNA copy numbers for *tax* were the highest in fraction 18 at 1.2 × 10^5^ (control) and 3.9 × 10^4^ (IR) RNA copies (Figure 3D). Similarly, *hbz* RNA levels were highest in fraction 18, quantifying at 4.4 × 10^5^ (control) and 1.8 × 10^5^ (IR), which is interesting as an increase in *hbz* RNA did not result in HBZ protein synthesis. Overall, these data indicate that viral proteins and RNA are present in different density EVs and that processed viral components are potentially more abundant in the highest density EVs, while unprocessed proteins in lower density EVs.

### Characterization of the Effects of EVs From HTLV-1-Infected Cells on Recipient Cells

**FIGURE 4:**
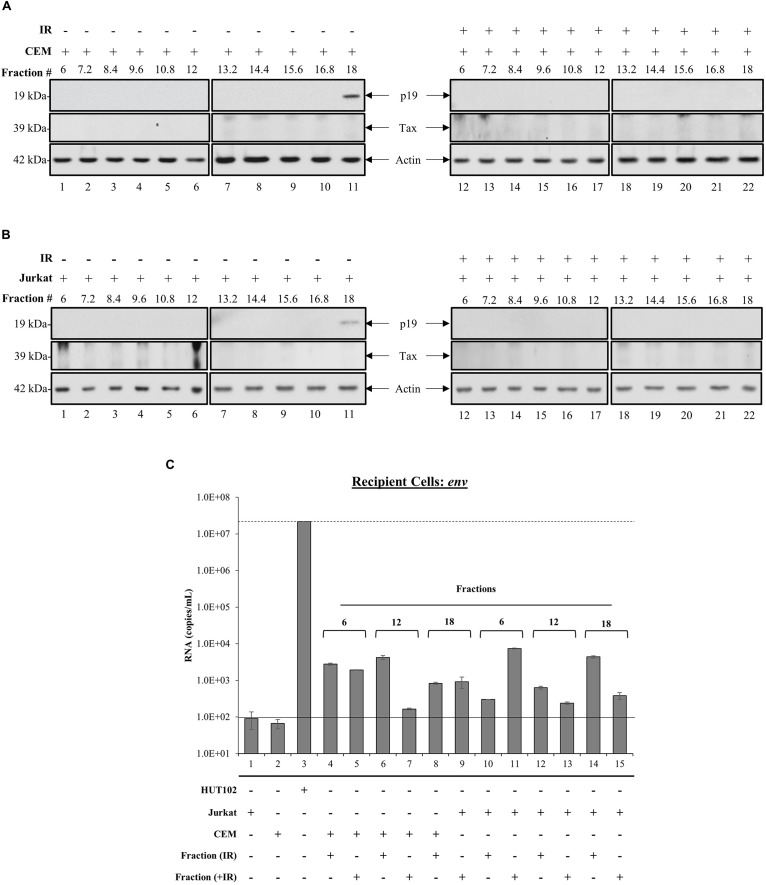
Treatment with HTLV-1 EVs does not generate HTLV-1 viral proteins and RNA in Recipient cells. CEM and Jurkat cells were treated with density fractions (6 to 18) of HUT102 EVs for 5 days. WB analysis was conducted on CEM **(A)** and Jurkat **(B)** cells treated with HUT102 control EV fractions (lanes 1–11) and HUT102 IR-treated EV fractions (lanes 12–22) for p19, Tax, and Actin. Selected lanes were taken from the same blot with identical exposure settings presented in the figure. **(C)** RT-qPCR was conducted on WCE from Jurkat, CEM, and HUT102 cells (controls; lanes 1, 2, and 3, respectively). Jurkat cells (±IR) treated with fractions 6 (lanes 4 and 5), 12 (lanes 6 and 7), and 18 (lanes 8 and 9), and CEM cells (±IR) treated with fractions 6 (lanes 10 and 11), 12 (lanes 12 and 13), and 18 (lanes 14 and 15) for the presence of *env* RNA. Negative controls levels were denoted by a solid line (-) and positive control levels for infection by a dashed line (—). We used fraction 6 as negative control for presence of virus; fraction 18 as a potential source of virus; and fraction 12 as a control for fraction 18, which contained EVs with Tax/gp61.

Whether EVs derived from HTLV-1-infected cells are capable of establishing a new infection is not known. However, the presence of viral proteins and RNAs in EVs from infected cells implies that they may be able to cause disease in recipient cells. We observed previously that fraction 18 contained a higher abundance of processed viral proteins (p19 and gp46) and RNA (*env*, *tax*, and *hbz*), which led us to hypothesize that this fraction may potentially be infectious. Therefore, we investigated the effects of all HTLV-1 EV fractions (±IR) on uninfected recipient T-cells (CEM and Jurkat). Recipient cells were harvested after 5 days and analyzed for the presence of viral RNA or proteins. Both p19 and Tax were not detected in CEM cells treated with EV fractions (lanes 1–22), except for the presence of p19 in fraction 18 (Figure 4A; lane 11). The results in Figure 4B, in which EV fractions from HTLV-1-infected cells were added to Jurkat recipient cells, demonstrated a trend similar to that observed in CEM recipient cells for all measured proteins. Interestingly, in both CEM and Jurkat cells, fraction 18 from IR treated HUT102 cells did not elicit the expression of p19 in recipients (Figures 4A,B; lane 22). Altogether, these data suggest that while fractions isolated from HTLV-1-infected cells contain viral proteins and RNA, only fraction 18 may have the potential to establish a new infection, and possibly includes both EVs and mature virions.

To further elucidate the functionality of fraction 18, we asked whether the observed p19 was due to carryover or instead novel viral replication and protein translation. The effects of each EV fraction (±IR) on uninfected recipient cells was assessed by RT-qPCR of *env* RNA levels in negative controls (Figure 4C; solid line) Jurkat (lane 1) and CEM cells (lane 2), positive control (Figure 4C; dashed line) HUT102 (lane 3); and recipient CEM/Jurkat cells treated with ±IR fractions 6, 12, and 18. Jurkat and CEM cells showed a background of 92 and 67 RNA copies, respectively (Figure 4C). Positive control HUT102 cells showed a baseline *env* RNA copy number of 2.2 × 10^7^, which served as a control for infection. Quantitation of Jurkat or CEM cells treated with EV fractions from HTLV-1-infected cells (±IR) resulted in RNA levels above negative controls (Figure 4C; lanes 1 and 2), but below positive control (Figure 4C; lane 3), regardless of IR treatment (Figure 4C; lanes 4–15). This data further validates that EVs from HTLV-1-infected cells do not cause a new round of infection, yet the EVs may be successful at delivering their cargo into recipient cells, as evident by baseline *env* RNA levels detected in all cells receiving EVs.

**FIGURE 5:**
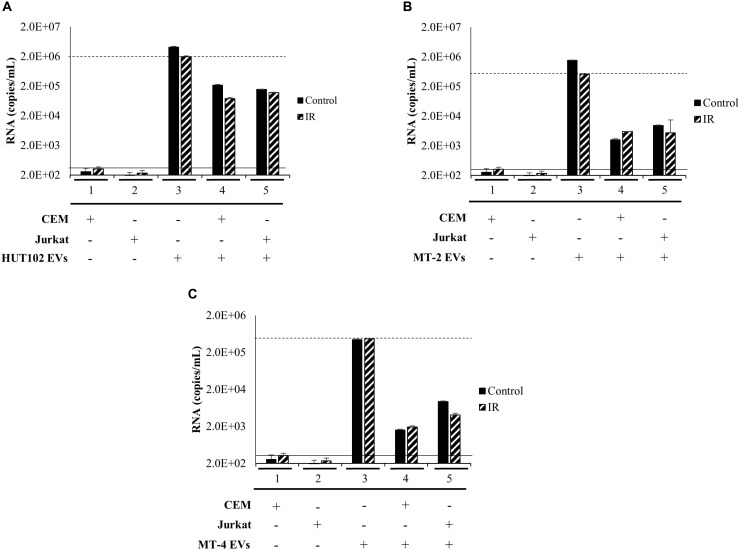
EVs isolated from HTLV-1-Infected Cells are not Infectious. T-cell lines (CEM and Jurkat) were cultured in EV-depleted media and incubated with EVs from HTLV-1-infected cells (HUT102 **(A)**, MT-2 **(B)**, or MT-4 **(C)**; ±IR) for 5 days. RT-qPCR for *env* RNA was performed for control CEM cells (lane 1), Jurkat cells (lane 2). Additionally, HTLV-1 EVs (HUT102, MT-2, or MT-4; lane 3) was also analyzed to determine relative RNA levels of the starting material. RT-qPCR quantitation of recipient CEM and Jurkat cells were analyzed to assess infectivity (lanes 4 and 5, respectively). Negative controls levels were denoted by a solid line (-) and positive control levels for infection by a dashed line (—).

We next sought to investigate whether a heterogeneous EV population, rather than those separated explicitly by a density gradient, may establish infection in uninfected T-cells, as measured by an increase in RNA levels above and beyond negative controls (Figures 5A–C; solid line) to a positive control (Figures 5A–C; dashed line). EVs from HTLV-1-infected HUT102, MT-2, and MT-4 donor cells (±IR) were enriched using NT080/082 and used for subsequent co-culture with recipient cells (CEM and Jurkat). Following incubation, recipient cells were harvested and analyzed by RT-qPCR (Figure 5). Not surprisingly, EVs from HUT102 cells did not result in an increase in *env* RNA copy numbers in recipient cells (lanes 4 and 5) compared to the amount of env RNA present in the EV input (Figure 5A; lane 3). Similar data was also observed for MT-2 (Figure 5B) and MT-4 (Figure 5C) EVs. Overall this data suggests that HTLV-1 EVs may not be infectious, however, EVs are capable of carrying viral RNA and/or proteins into the recipient/target cell.

### EVs From HTLV-1-Infected Cells Promote Cell-to-Cell Contact

**FIGURE 6:**
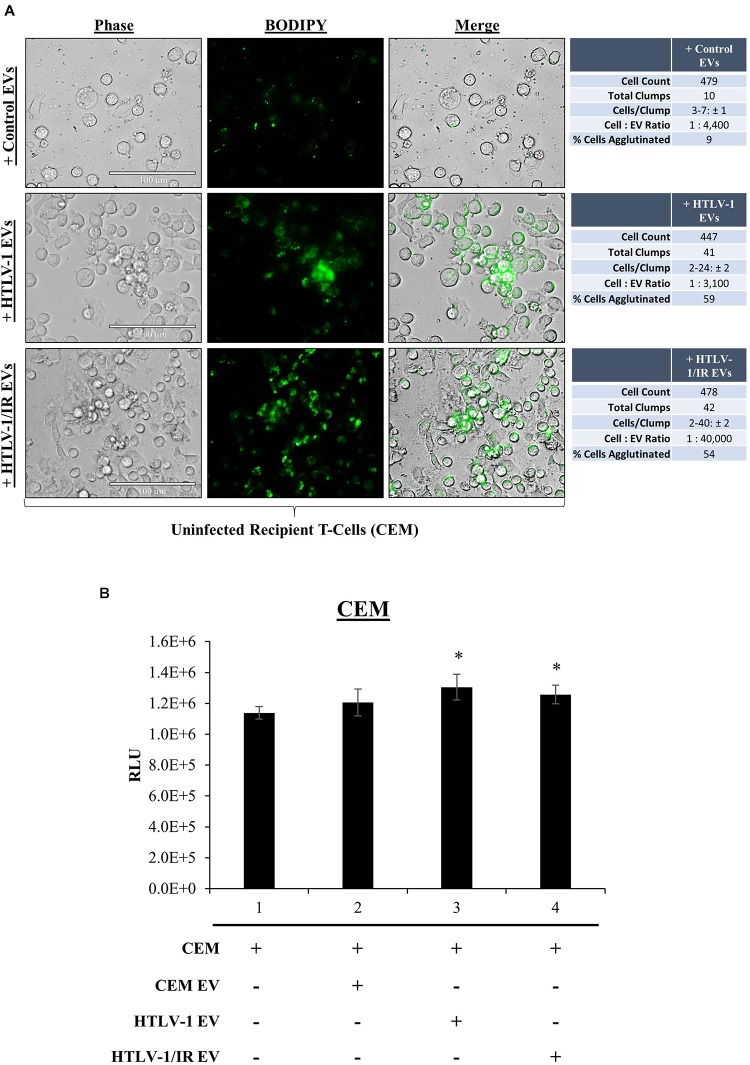
EVs from HTLV-1-Infected Cells Promote Cell-to-Cell Contact. **(A)** Uninfected recipient cells (CEM) in biological triplicate were cultured at 1 × 10^5^ cells/mL in a 100 μL well and treated with equal volumes of CEM EVs (4.43 × 10^8^ EVs; 1:4,400), HTLV-1 EVs (3.05 × 10^8^ EVs; 1:3,300), and HTLV-1/IR EVs (4.02 × 10^9^ EVs; 1:44,000) and allowed to incubate for 5 days prior to fluorescent microscopy analysis. Cellular aggregates were counted when Green Fluorescence signals from EVs was found associated with recipient cell membranes and cells were in direct contact with each other. Attributes recorded were number of aggregates, number of cells per aggregate, and number of total cells per field of view. EVs used in treatments were concentrated by ultracentrifugation at 100,000 × *g*. Margin of error (±) reported for Cells/Clump with a 95% confidence interval. **(B)** Cell viability of recipient cells (CEM; 5 × 10^5^ cells/mL, in biological triplicate) treated with EVs (5 × 10^9^ cells/mL) at a 1: 10,000 ratio. A two-tailed student *t*-test was used to evaluate statistical significance with a ^∗^*p*-value ≤ 0.05 indicating the level of significance.

We have previously shown that EVs have functional effects on recipients cells (Narayanan et al., 2013; Sampey et al., 2016; Anderson M.R. et al., 2018). Although our data suggest that HTLV-1 EVs may not be infectious in T-cells, *env* RNA was still detected in recipient cells, therefore we asked whether EVs may have functional effects on neighboring recipient cells (i.e., cargo delivery). During prior treatments of recipient cells with HTLV-1 EVs, increased cell aggregation was noted. We hypothesized that HTLV-1 EVs might promote increased cell-to-cell contact of recipient uninfected cells. It was first necessary to track HTLV-1 EVs to determine if they colocalized with recipient cells. EVs derived from donor CEM cells (Control EVs), HUT102 cells (HTLV-1 EVs), or HUT102 IR-activated cells (HTLV-1/IR EVs) were fluorescently labeled with BODIPY, and added to recipient CEM cells at EV concentrations normalized to volume to better resemble physiological conditions (Figure 6A). Twenty-four hours post-treatment, Control EVs localized sparsely in the extracellular medium, and minimally with recipient cells. Only 9% of all CEM cells were found agglutinated together. Aggregates did not seem to be EV-mediated and may represent background levels (Figure 6A; upper panel). Interestingly, HTLV-1 EVs (Figure 6A; middle panel) were found to colocalize with cell membranes of recipient cells agglutinated together resulting in a total of 59% of cell agglutination (only EV-mediated aggregates counted), and similarly for HTLV-1/IR EVs (Figure 6A; lower panel) at 54% agglutination. However, it was noted that HTLV-1/IR EVs caused a 2-fold increase in the maximum number of cells agglutinated (from 24 ± 2 to 40 ± 2 cells/clump). Additionally, cell viability of recipient cells was assessed up to 5 days of post-EV treatment and analysis revealed no detrimental effects on viability (Figure 6B). On the contrary, HTLV-1 EV treated recipient cells (lanes 3 and 4) showed significantly improved viability (*p*-value ≤ 0.05) when compared to untreated cells (Figure 6B; lane 1). These data suggest that HTLV-1 EVs promote cell-to-cell contact without decreasing cell viability, a finding that may be important for viral spread.

### Increased Cell-to-Cell Contact Promotes Potential Increase in Viral Spread

**FIGURE 7:**
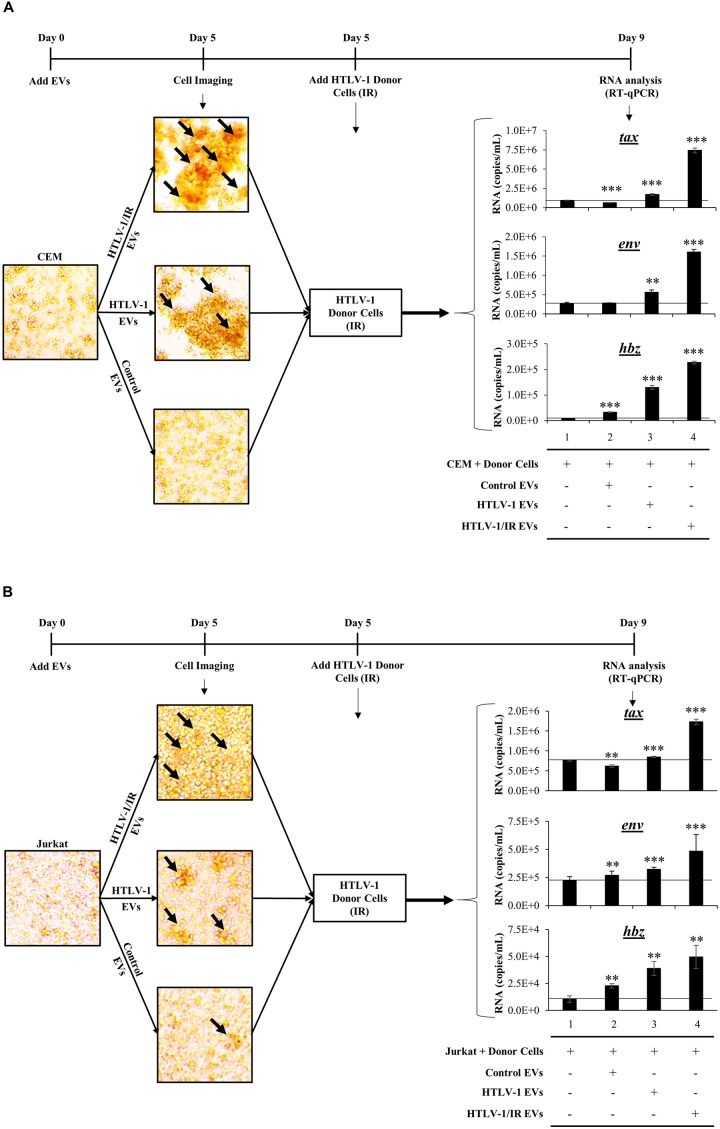
HTLV-1 EVs Promote Cell-to-Cell Contact and Increase Infectivity of HTLV-Infected Cells. Uninfected recipient CEM **(A)** and Jurkat **(B)** cells were cultured in biological triplicate in EV-depleted media with EVs from CEM cells (Control EVs), HUT102 cells (HTLV-1 EVs), and irradiated HUT102 cells (HTLV-1/IR EVs) at a ratio of 1 cell to 10,000 EVs for 5 days prior to microscopic analysis. Images are representative of three independent experiments. Following microscopy, irradiated HUT102 cells (HTLV-1 Donor Cells; 10 Gy) and fresh Exo-Free media were added to the culture at a ratio of 1:100 for 4 days. Subsequent RT-qPCR analysis was performed for the presence of *tax*, *env*, and *hbz*. A two-tailed student *t*-test was used to evaluate statistical significance with ^∗∗∗^*p*-values ≤ 0.001, *^∗∗^p*-values ≤ 0.01, and ^∗^*p*-values ≤ 0.05 indicating the level of significance.

We consistently observed increased agglutination of recipient CEM cells treated with HTLV-1 EVs normalized to volume. We next validated our observations by testing the effects of EVs on recipient cells by normalizing treatment to EVs concentration (1:10,000; Cell:EV ratio) at day 5 post-EV treatment, and by the use of multiple different uninfected recipient cells (i.e., CEM and Jurkat). Additionally, we asked whether the observed increased agglutination could also correlate to increased viral transcription on recipient cells. To test this, IR treated HUT102 donor cells were added at day 5 and cultured for 4 additional days prior to performing RT-qPCR analysis (Figure 7). In this scenario, IR treatment of HTLV-1-infected donor cells (which inhibits cellular replication) is the source of the viral spread.

Initial microscopic analysis revealed the highest agglutination levels in recipient cells when treated with HTLV-1/IR EVs, suggestive of high levels of cell-to-cell contact (Figure 7A; black arrows in the upper panel; larger clumps). HTLV-1 EVs (Figure 7A; middle panel) elicited a moderate amount of cell-to-cell contact between uninfected cells, while minimal contact was observed in the control EV treatment (Figure 7A; lower panel). Quantitation of HTLV-1 RNA by RT-qPCR indicated that *tax* RNA levels increased above background when pretreated with HTLV-1/IR EVs (Figure 7A; compare *tax* lanes 1–4) and to a lesser extent when treated with HTLV-1 EVs (compare *tax* lanes 1–3). Similar results were obtained for *env* and *hbz*. Overall, these data suggest that HTLV-1 EVs (±IR) promote increased levels of viral transcription in recipient CEM T-cells.

Jurkat recipient T-cells were also tested. They showed similar levels of cell agglutination with HTLV-1/IR and HTLV-1 EVs, and minimal levels with Control EVs (Figure 7B). Subsequently, HTLV-1 donor cells (HUT102, IR activated) were added and incubated as described for CEM cells. RT-qPCR analysis indicated that *tax* RNA levels increased above background when pretreated with HTLV-1/IR (Figure 7B; compare *tax* lanes 1–4) and HTLV-1 EVs (compare *tax* lanes 1–3), but decreased from the background when treated with Control EVs (compare *tax* lanes 1 and 2). RNA levels for *env* increased the most above background when treated with HTLV-1/IR EVs (*env* lane 4), to a lesser extent when treated with HTLV-1 EVs (*env* lane 3) and the least with Control EVs (*env* lane 2). Finally, *hbz* levels exhibited the highest increase above background when treated with HTLV-1/IR EVs (*hbz* lane 4), moderately with HTLV-1 EVs (*hbz* lane 3), and the least with Control EVs (*hbz* lane 2). These data suggest that HTLV-1 EVs (±IR) may have functional effects in priming distinct recipient T-cells for infection (i.e., Jurkat and CEM). Overall, these data suggest that HTLV-1 EVs promote cell-to-cell contact of uninfected recipient cells, which could potentially facilitate viral spread.

### Antibodies Against Specific Cellular Surface Receptors Inhibit Cell-to-Cell Contact

HTLV-1/IR EVs consistently promoted increased cell-to-cell contact, and CEM recipient cells were the most receptive to this effect. We, therefore, attempted to elucidate the mechanism by which EVs promote this phenotype. HTLV-1 transmission primarily occurs via cell-to-cell contact facilitated by several potential proteins involved in cell adhesion, such as CD45 (viral biofilm), ICAM-1, VCAM-1, and LFA-1 (cellular conduits and virological synapse), and Tax/Gag proteins (tunneling nanotubes) (Van Prooyen et al., 2010b; Gross and Thoma-Kress, 2016; Gross et al., 2016; Omsland et al., 2018). We hypothesized that HTLV-1/IR EVs could carry proteins involved in cell adhesion. Thus, neutralization with antibodies would potentially inhibit HTLV-1 EV-mediated cell-to-cell contact.

**FIGURE 8:**
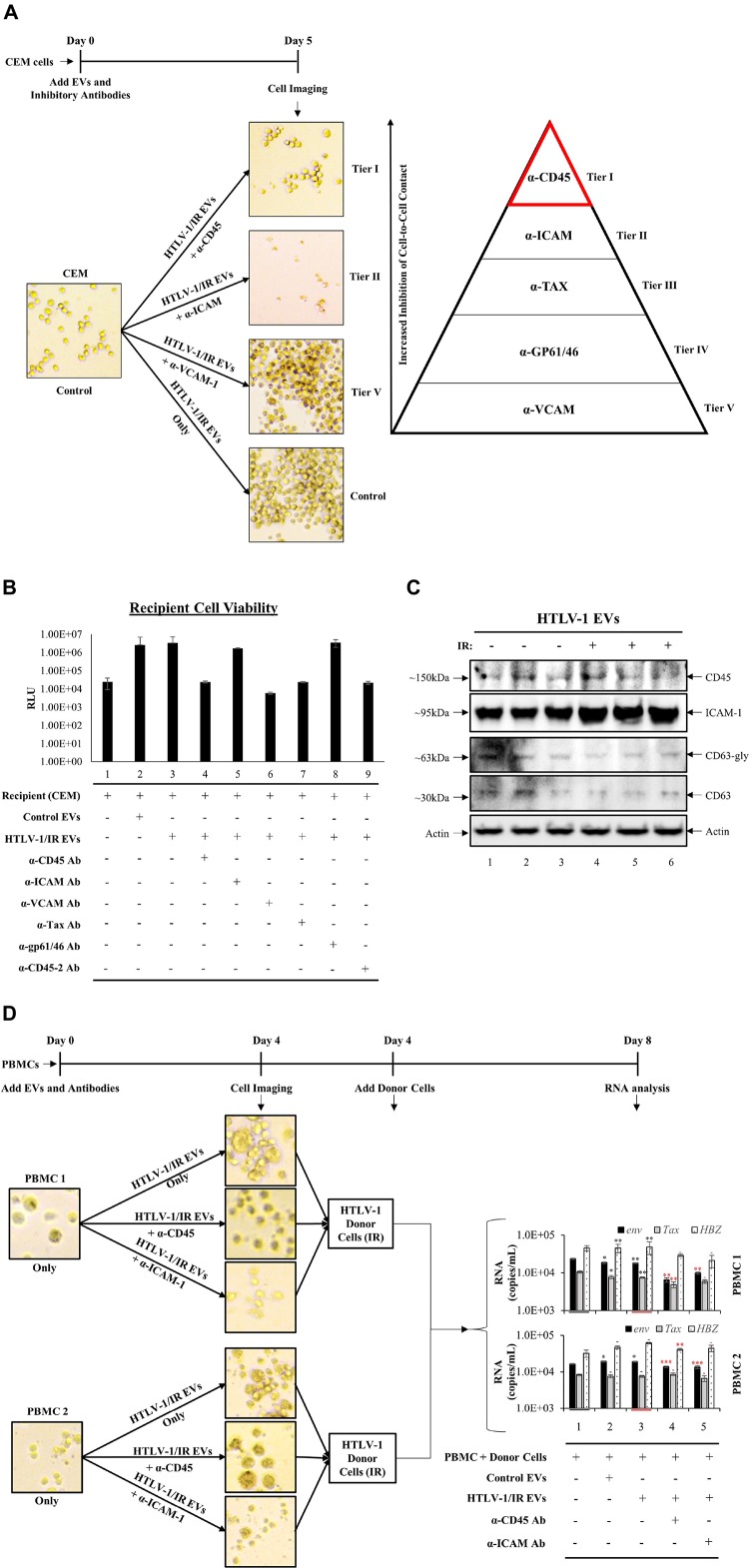
Antibodies against Specific Cellular Surface Receptors Inhibit Cell-to-Cell Contact. **(A)** CEM recipient cells, in biological triplicate, were treated with HTLV-1 EVs (1 cell: 10,000 EVs) and treated with antibodies at concentrations derived from titration (data not shown) for α-CD45 (0.2 μg/mL), α-ICAM-1 (20 μg/mL), α-VCAM-1 (20 μg/mL), α-Tax (7.5 μg/mL of three Tab antibodies; 1:100 dilution), α-gp61/46 (5 μL of 1:10 dilution; according to Palker et al., 1992; 45 μL/1 × 10^6^ cells/mL), and imaged at day 5. **(B)** Cell viability of CEM cells (5 × 10^5^ cells/mL) treated with HTLV-1/IR EVs (5 × 10^9^ cells/mL) and neutralizing antibodies at day 5 after treatment. **(C)** Western blot analysis for CD45, ICAM-1, CD63 and Actin was performed on HTLV-1 EVs (from HUT102 cells) enriched by NT080/082 from supernatants of 5 days old CEM cultures at 1 × 10^6^ cells/mL. **(D)** PBMCs were cultured for 3 days and with IL2 and PHA on day 0 and day 3 prior to treatment with HTLV-1/IR EVs (1 PBMC: 10,000 EVs) and α-CD45 and ICAM-1 for 4 days, and subsequent addition of IR treated donor cells (1 HUT102 cell: 100 PBMCs) and RT-qPCR analysis for *env*, *tax*, and *hbz* at day 8. Only 2 out of 3 PBMC experiments are shown. Black Asterisks (^∗^) are used to compare lanes 2 and 3 to lane 1, and red asterisks (^∗^) are used to compare lanes 4 and 5 to lane 3.

Recipient cells (CEM) were cultured in triplicate and treated with HTLV-1/IR EVs as shown in Figure 7. Simultaneously, neutralizing antibodies against CD45, ICAM-1, VCAM-1, Tax, or gp61/46 was added to test for cell-to-cell contact inhibition (data not shown) and one optimal concentration for each was used for blocking experiments. The most noticeable differences were observed using α-CD45 (Figure 8A; Tier I) and α-ICAM-1 (Figure 8A; Tier II) which prevented EV-mediated cell agglutination, whereas α-VCAM-1 (Figure 8A; Tier V) had no effect against EV-mediated cell-to-cell contact. Out of the two antibodies that effectively prevented cell-to-cell contact, α-CD45 treated cells showed morphology that resembled untreated CEM cells. However, although addition of α-ICAM prevented agglutination, cell morphology was altered (smaller size) and the pH of the media remained unchanged after 5 days, potentially suggesting an either lowering of intracellular ATP levels or a general cellular toxicity. Use of α-Tax (Tier III) and α-gp61/46 (Tier IV) antibodies only partially inhibited agglutination and, therefore, were not considered as primary proteins responsible for HTLV-1 EV mediated cell-to-cell contact (data not shown). Overall, the data suggest that CD45 is a prime candidate in the mediation of cell-to-cell contact in HTLV-1 infection. However, it was still unclear whether CD45 protein was on the surface of the EV or on the recipient cell membrane.

Next, it was important to evaluate the viability of antibody treated cells, especially since antibodies may concomitantly inhibit normal cellular process leading to cell death. Additionally, viral transmission requires viable cells for effective viral spread. Therefore, we validated recipient cell viability using five neutralizing antibodies (and a duplicate α-CD45 antibody). Cell viability analyses revealed no significant change in cell survival after antibody treatment (Figure 8B), as a two-tailed *t*-test confirmed no significance between changes in viability when compared to either untreated CEM cells (lane 1) or HTLV-1/IR EV treatment (lane 3). These data suggest that the inhibition of cell-to-cell contact by α-CD45 is potentially limited to interactions induced by EVs, and not physiologically necessary interactions in the tested *in vitro* settings.

We next explored whether CD45 and/or ICAM-1 are present in HTLV-1 EVs (±IR), hence contributing to viral spread. Supernatant from 5-day old cultures in biological triplicate were separated and enriched for EVs by NT080/082 (Figure 8C). WB analysis revealed trace amounts of CD45 in HTLV-1 EVs (Figure 8C; lanes 1 and 3) and no significant difference in HTLV-1/IR EVs (Figure 8C; lanes 4–6). ICAM-1 was detected in HTLV-1 EVs and HTLV-1/IR EVs (lanes 1–6). These data show the potential presence of CD45 and ICAM-1 in HTLV-1 EVs (±IR), supporting prior observations about their potential role in mediating increased levels of cell-to-cell contact.

Finally, two distinct donor PBMC cultures were treated with IL-2 and PHA for 3 days prior to treatment with HTLV-1/IR EVs (as described previously; 1:10,000; Cell:EV ratio) and neutralizing antibodies (α-CD45 or α-ICAM-1) were added in fresh EV-depleted RPMI media for 4 days. PBMCs treated with HTLV-1/IR EVs showed increased cell agglutination (Figure 8D; upper panel for both PBMCs), which was blocked by α-CD45 (Figure 8D; middle panel) and α-ICAM-1 treatment (Figure 8D; lower panel). Next, at day 4, HTLV-1 donor cells (IR; 10 Gy) were added as the source of virus at a ratio of 1 donor cell to 100 recipient cells (1:100). At day 8, RT-qPCR analysis revealed that *env*, *tax*, and *hbz* RNA levels were present in PBMCs above the background (starting material; lane 1). Addition of HTLV-1/IR EVs did not alter RNA levels dramatically. However, addition of α-CD45 antibody resulted in a decrease in *env* and *tax* levels for PBMC 1; and in *env* and *hbz* for PBMC 2 (compare lane 3 and 4). Addition of α-ICAM antibody resulted in a decrease in *env* RNA levels for both PBMC 1 and 2 (compare lane 3 and 5). Collectively, these data suggest that CD45 may be an important molecular target in EV-mediated cell-to-cell contact, followed by ICAM-1, since their inhibition potentially suppresses viral transmission in PBMCs.

### EVs From HTLV-1-Infected Cells Promote Viral RNA Transcription and DNA Integration Across Multiple Tissues *in vivo*

**FIGURE 9:**
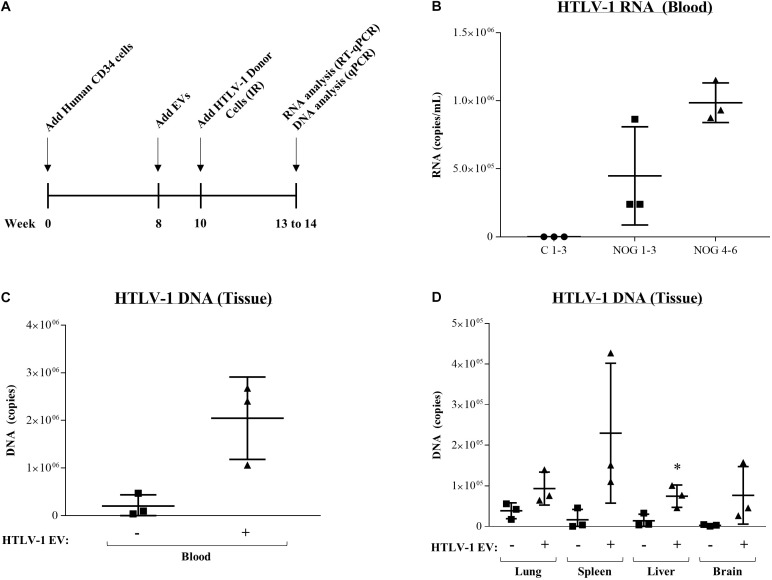
EVs from HTLV-1-infected cells increase viral RNA and DNA in NOG mouse model. **(A)** NOG mice received human CD34 cells (8 weeks), followed by control EV or HTLV-1 EV injection (HUT102; 6×, 10 μg for 2 weeks), and subsequent treatment with IR activated HTLV-1 donor cells (50 million) for 3 weeks. IR treatment of HTLV-1-infected cells (inhibition of cellular replication) is the source of the viral spread. **(B)** RT-qPCR of viral RNA from blood of NOG mice (*n* = 9) that received no treatment (C 1–3; denoted by a circle), Control EV treatment (NOG 1–3; denoted by a square, and HTLV-1 EVs (NOG 4–6; denoted by a triangle). **(C)** Presence of HTLV-1 DNA (using PCR for *env* region) from the blood of NOG mice. **(D)** Analysis of HTLV-1 DNA (*env*) from NOG mice from Lung, Spleen, Liver, and Brain. A two-tailed student *t*-test was used to evaluate statistical significance with ^∗∗^*p*-values ≤ 0.01 and *^∗^p*-values ≤ 0.05 indicating the level of significance compared to control.

Finally, to test the effect of EVs *in vivo*, we utilized HTLV-1-infected NOD/Shi-scid/IL-2Rγc null (NOG) mice, which have shown successful viral spread and ATLL development *in vivo* (Tezuka et al., 2014, 2018). NOG mice received human CD34^+^ cells (8 weeks), followed by HTLV-1 EV injection (HUT102; 6×, 10 μg each for 2 weeks), and subsequent treatment with IR activated HTLV-1 donor cells (50 million cells) for 3 weeks (Figure 9A). As previously described, IR treatment of HTLV-1-infected cells is the source of the viral spread. NOG mice were treated with EVs from HTLV-1-infected cells or EVs from uninfected control cells followed by injection of IR activated HTLV-1 donor cells. All mice exposed to HTLV-1-infected cells (NOG 1–6) showed upregulation of HTLV-1 RNA in blood cells (Figure 9B). Interestingly, NOG mice that received HTLV-1 EVs showed enhanced viral replication compared to mice which received Control EVs (Figure 9B; compare NOG 1–3 vs. NOG 4–6). Uninfected and untreated NOG mice controls (Figure 9B; C1-3) served as a baseline for infection. We then examined the presence of HTLV-1 DNA in various tissues. Data in Figure 9C indicates a 90% increase in HTLV-1 DNA, which was detected in the blood of animals that received HTLV-1 EV treatment compared to control EV treatment. HTLV-1 DNA levels were also increased in other tissues (Figure 9D) after HTLV-1 EV treatment, such as in lung (59% increase), spleen (93% increase), liver (81% increase; *p*-value ≤ 0.05), and brain (97% increase). Therefore, animals which received HTLV-1 EVs showed higher viral load in all tissues, including the brain, suggesting the virus was potentially able to expand into the anatomically privileged tissues. Collectively, these data indicate that EVs may contribute to viral spread *in vivo*, by promoting cell-to-cell contact and in turn potentiating the infection.

## Discussion

Our data validate findings that HTLV-1-infected cells secrete EVs that contain Tax protein. The presence of Tax in EVs are in line with previous findings of EV associated Tax in cell lines, PBMCs, and CSF from HAM/TSP patients (Jaworski et al., 2014a; El-Saghir et al., 2016; Barclay et al., 2017a; Anderson M.R. et al., 2018; Otaguiri et al., 2018). Moreover, they show that an IR treated infected cell may potentially contain significantly higher levels of intracellular Tax. Tax levels could contribute to cellular events related to EV biogenesis, such as release, as well as other related machineries including control of autophagy. IR treated infected cells also secreted higher numbers of EVs that may contain more Tax, either as free Tax, or EVs that originate from multiple sources (i.e., Golgi vs. MVBs). Future experiments will better define the nature of these Tax containing entities.

Tax is the main oncoprotein in HTLV-1, therefore its association with EVs may have potential effects in perpetuating Tax-mediated pathogenesis, including the induction of NF-κB in recipient cells which may promote the development of ATLL (Azimi et al., 1998; de la Fuente et al., 2000; Azran et al., 2004). Additionally, expression of EV-associated Tax has been found consistently in HAM/TSP patients with functional roles such as overexpression of activated CD8^+^ Cytotoxic T-cells (Anderson M.R. et al., 2018). This overexpression may have direct implications in the development of HAM/TSP. We expand on previous work by showing that IR treatment may have modulatory effects on Tax-containing EVs (Figure 1). We found that IR not only increases the association of Tax with EVs, but it also causes an upregulation in the amount of EVs secreted from HTLV-1-infected cells. It is important to note that IR is a well-characterized stress inducer (Bartkova et al., 2005; Gorgoulis et al., 2005) and we have previously shown that it can activate LTR gene expression of HIV-1 (Iordanskiy et al., 2015). Interestingly, here we observed IR activates both 5′LTR and 3′LTR transcription in multiple different HTLV-1-infected cell lines as shown by increases in *tax*, *env*, and *hbz* gene expression (Figure 2). Interestingly, the increase in transcription was higher for the 3′LTR (∼2.5-log), since there was complete suppression of the 3′LTR prior to IR activation. In contrast, the 5′LTR activation was not as dramatic, since there were originally higher background levels of basal transcription from this promoter. Therefore, the use of IR to potentially reactivate HTLV-1 may likewise stimulate the increased production of EVs and packaging of Tax and other viral products into these EVs. Increased levels of Tax-containing EVs may thereby highly impact recipient cells during pathogenesis.

We next sought to characterize whether specific EV types were responsible for carrying select HTLV-1 cargo and whether such EVs were infectious. For the past several years, our lab has focused on optimizing the isolation of EVs from multiple different viral infection settings, including HTLV-1 (Jaworski et al., 2014a; Barclay et al., 2017a), HIV-1 (Narayanan et al., 2013; Jaworski et al., 2014b; Sampey et al., 2016; Barclay et al., 2017b; DeMarino et al., 2018), Ebola virus (Pleet et al., 2016, 2018b), and Rift Valley Fever virus (Ahsan et al., 2016). Separation of EVs by density through the use of Iodixanol gradients has been described previously as a method to reliably separate EVs away from viruses such as HIV-1 (DeMarino et al., 2018). Here, we found that HTLV-1 processed proteins, such as envelope, settled in the highest density fraction (#18), suggesting potential presence of either virus or EVs that contained virus (Figure 3). Fractions 7.2 to 13 contained mostly unprocessed proteins, such as gp61, and fraction 6 contained trace levels of gp46. Overall, this data allows an initial categorization of HTLV-1 EVs into three groups: large density EV complex (fraction 18), lower density EV complex (fraction 7.2 to 13), and potentially ≤35 nm EVs or free protein complexes (fraction 6). However, upon treatment of recipient T-cells with each EV fraction, neither fraction was able to show infection in recipient cells. Only fraction 18 resulted in a band for the Gag p19 HTLV-1, although we cannot formally discard the possibility that the p19 was carried over from NTs used for concentrating the fractions prior to their addition to cells (Figure 4). The use of all EVs types, as opposed to those separated by Iodixanol gradients, on recipient cells also resulted in no infection, validating the lack of infectivity of HTLV-1 EVs (Figure 5). It is important to note that the amount of viral protein or RNA in recipient cells was 1 to 3 logs less than starting NT concentrated material, suggesting that recipient cells might be processing the cargo of the delivered EVs.

Even though HTLV-1 EVs were not infectious, they promoted agglutinated phenotypes through cell-to-cell contact by direct interaction with uninfected recipient cell membranes (Figure 6). Here, we observed that HTLV-1 viral spread was most efficient when the recipient cells were primed by HTLV-1 EVs (±IR) followed by the addition of infected donor cells (Figure 7). This was true for all recipient cells as HTLV-1 RNA levels on recipient cells were consistently higher after HTLV-1 EV treatment. Interestingly, CEM recipient cells showed significant clustering of cells indicative of cell-to-cell contact mediated by HTLV-1 EVs (±IR). In this scenario, where the uninfected cells are agglutinated and an infected cell is in close proximity, the chances of viral spread may increase via known mechanisms of viral transmission, such as virological synapse, viral biofilm, cellular conduits, and/or tunneling nanotubes.

We also have performed similar experiments in monocyte U937 cells (Supplementary Figure S2). Microscopic analysis revealed higher numbers of cell aggregates post-, pre-treatment with HTLV-1/IR EVs (upper panel), followed by HTLV-1 EVs (middle panel), and no noticeable clustering with Control EVs (lower panel). To determine if these phenotypes correlated with changes in HTLV-1 RNA levels, RT-qPCR was performed. *Hbz* RNA levels were increased by 2-fold HTLV-1 EVs and 4-fold with HTLV-1/IR EVs in monocytes. However, *tax* RNA levels did not increase with either treatment, indicating that there might be an inherent difference between T-cell and monocyte infection of HTLV-1. Along, these lines, it has previously been shown that monocytes do not support *in vitro* HTLV-1 infection and can undergo SAMHD1-dependent apoptosis through abortive cDNA synthesis (Sze et al., 2013). Although our EV results on monocyte show agglutination, future experiments will determine whether there is low levels of full cDNA synthesis, resulting in low copy number integrations and regulation with the 3′LTR resulting in *hbz* RNA transcripts.

Our data suggest that CD45 (or its binding partner CD43) in HTLV-1 EVs is a potential mediator between HTLV-1 donor cells and recipient T-cells (Figure 8), allowing the assumption that HTLV-1 EVs promote viral transmission via viral biofilm formation or use of ICAM-1 through virological synapse. Altogether, these data suggest that HTLV-1 EVs may amplify viral spread via upregulated cell-to-cell contact between uninfected cells.

We then sought to further validate the *in vitro* increase in potential viral spread, through an increase in cell-to-cell contact, in an *in vivo* humanized mouse model. The NOG mice treated with HTLV-1 EVs showed an increase in blood RNA levels (3 out of 3) compared to mice that received control EV treatment (Figure 9B). Moreover, HTLV-1 EVs consistently caused an increase in HTLV-1 DNA copies in all tissues from animals treated with HTLV-1 EVs (Figures 9C,D). Most interestingly, HTLV-1 DNA levels in anatomically privileged tissues, such as the brain, were drastically elevated in animals treated with HTLV-1 EVs versus below background levels in mice that received only Control EVs. These data suggest that HTLV-1 EVs not only promote viral spread via clustering of uninfected cells, but they potentially allow for viral spread across anatomical barriers, pointing toward evidence of the potential importance of EVs in CNS related HTLV-1 pathogenesis (i.e., HAM/TSP), as well as in the establishment of latent viral reservoirs.

**FIGURE 10:**
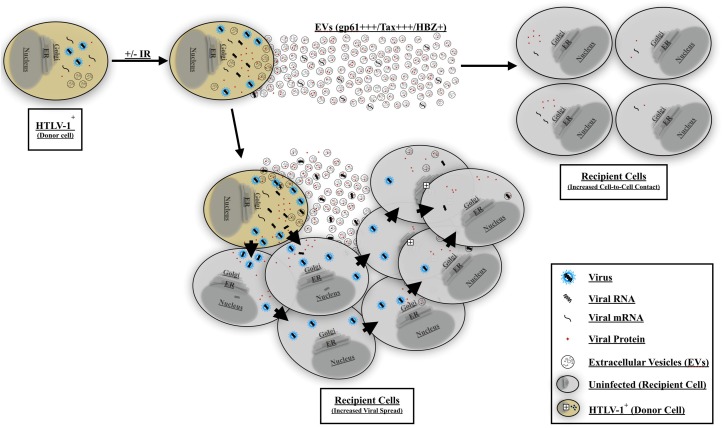
Proposed model for EV-mediated HTLV-1 viral spread and pathogenesis. HTLV-1 EVs carrying viral cargo are released and prime uninfected recipient cells. The recipient cells become activated and/or migrate toward the HTLV-1-infected donor cell thereby promoting increased cell-to-cell contact. HTLV-1 donor cells may then transmit the virus to adjacent recipient cells and facilitate viral spread. Viral spread may occur via viral biofilm, virological synapse, cellular conduits, or TNT formation.

Finally, to better explain the observed EV-mediated phenotype in HTLV-1 infection, we propose a two-step model for viral spread. Our current model indicates that EVs from HTLV-1-infected cells (±IR) may play an important role in priming uninfected recipient cells for infection (Figure 10). Following EV release from HTLV-1-infected donor cells, these EVs may reach nearby uninfected recipient cells (especially in tissues), activate (potentially through NF-κB), and/or attract cells toward infected donor cells. Subsequently, following the migration of the uninfected recipient cells, cell-to-cell contact may occur. Neighboring cells may form a viral biofilm and/or virological synapse by which virus may pass to the adjacent recipient cells (Igakura et al., 2003; Majorovits et al., 2008; Nejmeddine et al., 2009; Gross and Thoma-Kress, 2016; Omsland et al., 2018). Other potential mechanisms of viral spread may also include formation of cellular conduit, and/or tunneling nanotube (TNT) (Pais-Correia et al., 2010; Van Prooyen et al., 2010a, b; Pise-Masison et al., 2014; Omsland et al., 2018) and other viral and cellular proteins (Van Prooyen et al., 2010a; Chevalier et al., 2014; Gross et al., 2016; Omsland et al., 2018). Ongoing and future experiments should aim to further elucidate the mechanisms of EV mediated viral spread and HTLV-1 pathogenesis, specifically studies should focus on the type of EV involved and the associated set of proteins that mediate cell-to-cell contact.

## Materials and Methods

### Cells

Human T-cell leukemia virus-1-infected cell lines HUT102, MT-2, and MT-4, uninfected T-cell (CEM and Jurkat), and promonocytic (U937) cell lines were cultured in RPMI-1640 media (Quality Biological) supplemented with 10% heat-inactivated exosome-free fetal bovine serum (FBS), 2 mM L-glutamine, 100 U/mL penicillin, and 100 μg/mL streptomycin for 5 days (at 37°C and 5% CO_2_) before harvesting for downstream experiments. For IR experiments, all cells were plated at a density of 1 × 10^6^ cells/mL, while co-culture experiments were plated at a density of 5 × 10^6^ cells/mL at day 0. Peripheral blood mononuclear cells (PBMCs) were isolated from peripheral blood from healthy, anonymous donors using Ficoll gradient centrifugation and then expanded in medium containing 1 μg/mL PHA-L and 30 or 50 IU/mL rhIL-2. After 2 days of cultivation the cells were washed and then cultured in the medium 30 IU/mL rhIL-2 without PHA-L.

### X-Ray Irradiation

All ionizing radiation (IR) treatments were performed in a RS-2000 X-Ray Irradiator (Rad Source, Suwanee, GA, United States) for a total radiation dose of 10 Gy per *in vitro* experiment. Samples were irradiated at day 0 and used for NOG mouse studies or allowed to incubate for 5 days prior to harvesting for downstream assays.

### EV Enrichment Using Nanotrap Particles (NTs)

Nanotrap particles (Ceres Nanosciences, Inc.) were used to enrich EVs from low volume, cell-free supernatant samples (1 mL) as previously described (Narayanan et al., 2013; Ahsan et al., 2016; Pleet et al., 2016, 2018b; Sampey et al., 2016; Barclay et al., 2017b; DeMarino et al., 2018). Briefly, a 30% slurry of NT082 (Ceres #CN2010), NT080 (Ceres #CN1030), and 1× Phosphate Buffered Saline (PBS) were combined, and 30 μL was added to 1 mL of each sample supernatant. The samples were then rotated overnight at 4°C to capture EVs. The resulting pellet was washed once with 1× PBS and used for downstream assays.

### Western Blot Analysis

Cell were harvested, pelleted, washed once in 1× PBS and resuspended in lysis buffer [50 mM Tris–HCl (pH 7.5), 120 mM NaCl, 5 mM EDTA, 0.5% Non-idet P-40, 50 mM NaF, 0.2 mM Na3VO4, 1 mM DTT, and 1 complete protease inhibitor mixture tablet/50 mL (Roche Applied Science)]. Lysate suspensions were incubated on ice for 20–30 min and vortexed at intervals of 5 min, followed by centrifugation at 10,000 × *g* at 4°C for 10 min to remove cell debris. Protein concentrations from lysates were quantified using Bradford protein assay (BIO-RAD).

For Western blot analysis, cell lysates (10–30 μg) were resuspended in 10 μL Laemmli buffer, heated at 95°C for 3 min, and loaded onto a 4–20% Tris–glycine SDS gel. Nanotrap particle pellets were resuspended in 10 μL Laemmli buffer, heated at 95°C for 3 min and vortexed repeatedly until fully resuspended (3 to 4 cycles). Material eluted from the Nanotraps were then loaded onto a 4–20% Tris–glycine gel. Transfer of proteins was done overnight at 50 mA onto PVDF membranes (Millipore). Membranes were blocked in 5% skim milk in 1× PBS containing 0.1% Tween 20 for 30 min at 4°C, then incubated overnight at 4°C with primary antibody at manufacturer recommended dilutions. Antibodies for protein targets include α-Tax (Tax antibodies 169, 170, and 171, monoclonal mouse; generous gift of Dr. Scott Gitlin, University of Michigan) (Jaworski et al., 2014a), α-p19 (Santa Cruz Biotechnology, sc-1665), α-gp61/46 (NIH AIDS Reagent Program Cat. #1578), 4D4-F3 α-HBZ (monoclonal mouse; generous gift from Dr. Roberto S. Accolla), α-CD45 (Santa Cruz Biotechnology, sc-1123), α-ICAM-1 (Santa Cruz Biotechnology, sc-8439), α-p53 (Abcam; ab32389), α-Cytochrome c (Santa Cruz Biotechnology, sc-13560), α-CD63 (Santa Cruz Biotechnology, sc-365604), and α-Actin (Abcam; ab469900). The next day, membranes were washed and incubated with the appropriate HRP-conjugated secondary antibody for 2 h at 4°C and developed using Clarity or Clarity Max Western ECL Substrate (BIO-RAD). Luminescence was imaged on a ChemiDoc Touch Imaging System (BIO-RAD). Raw densitometry counts were obtained using ImageJ software and by subtracting the background of each membrane. Data was normalized to Actin inputs for each protein of interest.

### ZetaView Nanoparticle Tracking Analysis (NTA)

Nanotracking analysis for EV quantification and sizing was performed using ZetaView^®^ Z-NTA (Particle Metrix) with its corresponding software (ZetaView 8.04.02). Calibration was performed using 100 nm polystyrene nanostandard particles (Applied Microspheres) prior to sample readings at a sensitivity of 65 and a minimum brightness of 20. Instrument parameters were set as previously described (DeMarino et al., 2018; Pleet et al., 2018b) for each reading. For each sample, 1 mL of sample diluted in DI water was loaded into the cell, followed by measurements of size and concentration of particles at 11 unique positions throughout the cell in three independent reads. After automated analysis and removal of any outliers, the mode diameter size and the concentration of the sample were calculated by the machine software. Measurement data from the ZetaView were analyzed using the corresponding manufacturer software, and raw data were plotted using Microsoft Excel 2016. The mode size of particles detected is presented in our data.

### EV Tracking and Fluorescent Microscopy

Between 50 and 80 μLs ultracentrifuged (at 100,000 × *g*) HUT102 cell supernatants (from 5-day old culture) at approximately 1 × 10^9^ EVs/mL were mixed with 1.5 μL of BODIPY^TM^ 493/503 (Cat. # D3922; Invitrogen^TM^) to label EVs for 30 min at 37°C, and run on a Pharmacia G-50 spin column (1 mL bed volume in PBS buffer; 2000 rpm/2 min; Sorval RT6000D) to remove unbound dye. Resulting BODIPY EVs were quantified again by ZetaView and added onto recipient cells normalized to volume in biological triplicate. Treated cells were analyzed with an EVOS-FL-Auto microscope (Life Technologies), under 20× and 40× magnification with phase objective and fluorescence.

### RNA Isolation, Generation of cDNA, and Real-Time qPCR

For analysis of HTLV-1 RNA, total RNA was isolated from either cell pellets of Nanotrap pellets. TRIzol Reagent (Invitrogen) was used according to manufacturer’s protocol. Total RNA was quantitated by a NanoDrop 1000 Spectrophotometer (Thermo Scientific) and used to generate cDNA with the GoScript Reverse Transcription System (Promega) using Oligo (dT). Next, RT-qPCR analysis was performed using 2 μL aliquots of undiluted cDNA, SYBR Green master mix (Bio-Rad), and specific primer sets were used: HTLV-1 *env* (env-Reverse 5′-CCA TCG TTA GCG CTT CCA GCC CC-3′, Tm = 64.4°C; env-Forward 5′-CGG GAT CCT AGC GTG GGA ACA GGT-3′, Tm = 64.5°C), *tax* (tax-Reverse 5′- AAC ACG TAG ACT GGG TAT CC-3′, Tm = 53.6°C; tax-Forward 5′- ATC CCG TGG AGA CTC CTC AA-3′, Tm = 57.6°C), and *hbz* (hbz-Reverse 5′-TGA CAC AGG CAA GCA TCG-3′, Tm = 55.7°C; hbz-Forward 5′-AGA ACG CGA CTC AAC CGG-3′, Tm = 57.8°C). Serial dilutions of DNA from HUT102 cells were used as quantitative standards. PCR conditions were as follows: for *env* primers 50°C for 2 min, 95°C for 2.5 min, then 40 cycles of: 95°C for 15 s, 64°C for 40 s; for *tax* primers 50°C for 2 min, 95°C for 2 min, Then 40 cycles of: 95°C for 15 s, 51°C for 20 s, and 72°C for 40 s; and *hbz*: 50°C for 2 min, 95°C for 2 min, Then 40 cycles of: 95°C for 15 s, 65.7°C for 20 s, and 72°C for 40 s. For RT-qPCR of *gapdh*, 2 μL aliquots of undiluted cDNA, using iQ Supermix (Bio-Rad), and specific set of primers sets and probes were used: cellular *gapdh (gapdh*-Reverse 5′-GAA GGT GAA GGT CGG AGT CAA C-3′, Tm = 57.5°C; *gapdh*-Forward 5′-CAG AGT TAA AAG CAG CCC TGG T-3′ Tm = 57.5°C; and *gapdh*-Probe 5′-/56-FAM/TTT GGT CGT ATT GGG CGC CT/36-TAMSP/-3′, Tm = 59.8°C). PCR conditions were as follows: for *gapdh* primers and probe 50°C for 2 min, 95°C for 2 min, then 39 cycles of: 95°C for 15 s, 50°C for 30 s. The quantification of samples was determined based on cycle threshold (Ct) values relative to the standard curve for each plate. Reactions were carried out in triplicate using the Bio-Rad CFX96 Real-Time System.

### ExoMAX EV Enrichment and Density Gradient Separation

HUT102 cells were seeded at a density of 1 × 10^6^ cells/mL in 30 mL of EV-depleted media and subjected to treatment (10 Gy). Following a 5 days incubation, supernatants were harvested using centrifugation (10,000 × *g* for 10 min) and EVs were precipitated by incubation with ExoMAX^TM^ reagent (1:1 ratio to sample volume; SBI) at 4°C overnight. Samples were then centrifuged (1,500 × *g* for 30 min) and resuspended in 300 μL of 1× PBS and loaded onto an Iodixanol gradient (OptiPrep^TM^; Sigma; 11 density fractions ranging from 6 to 18%). Separation of EVs into each fraction was accomplished by ultracentrifugation (24,100 × *g* for 90 min) in a SW41 Ti rotor (Beckman). Each fraction was collected into clean microcentrifuge tubes and EVs were concentrated with an NT080/082/086 particle mixture overnight as described above and used for downstream assays.

### Co-culture Treatments

Recipient cells (CEM, Jurkat, and U937) were incubated with corresponding EVs at a 1:10,000 ratio (5 × 10^6^ cells: 5 × 10^10^ EVs ratio) for 5 days. At day 5, HTLV-1 donor cells (HUT102) were subjected to IR (10 Gy) and co-cultured with the EV-treated, uninfected recipient cells at a ratio of 1:100 (5 × 10^4^ HTLV-1 donor cell: 5 × 10^6^ uninfected recipient cell ratio) and allowed to incubate for an additional 4 days prior to harvesting for downstream assays.

### Cell Contact Inhibition Assay

Antibodies against proteins involved in cell-to-cell contact were used at a final concentration of 0.2 μg/mL for α-CD45 (Santa Cruz Biotechnology, sc-1123), 0.2 μg/mL for the second α-CD45-2 (Santa Cruz Biotechnology, sc-53666), 20 μg/mL for α-ICAM-1 (Santa Cruz Biotechnology, sc-8439), 20 μg/mL for α-VCAM-1 (Santa Cruz Biotechnology, sc-13160), 0.12 μg/mL for α-Tax (1:100 dilution from a 7.5 μg/mL stock of a mix of Tax antibodies 169, 170, and 171, monoclonal mouse), and 1:10 dilution for α-gp61/46 (NIH AIDS Reagents Program; Cat. #1578).

### Cell Viability Assay

Cells were cultured in technical triplicates (i.e., 1 × 10^6^ cells/mL or at 5 × 10^5^ cells/mL) in 100 μL of fresh EV-depleted RPMI media in a 96 well assay plate (Corning Inc.; Cat#: 3610) and treated with HTLV-1 EVs as specified for each experiment. Antibodies were used to treat recipient cells to test for recipient cell viability at the concentrations specified above. Cells were allowed to incubate for zero or 5 days and CellTiter-Glo reagent (Promega; Cat#: G7572) was used at a 1:1 ratio to detect cell viability on a GloMax Explorer (Promega). EV-depleted RPMI media alone was cultured and used as background.

### Animal Model

NOD/Shi-scid/IL-2Rgc null (NOG) female pregnant mice were obtained from Jackson labs. NOG pups received human CD34^+^ cells (fetal liver embryonic cells; 500,000) 2 days post-initial IR treatment. Humanized animals (8–10 weeks) were treated with total EVs from HUT102 cells, 6 times over 2 weeks (10 μg in 200 μl PBS at intraperitoneal injection each). The animals were then injected with IR treated HTLV-1 donor cells (50 million, intraperitoneally) and kept for 3 weeks. IR treatment stops the replication of infected cells and allows for viral spread into new human cells in the animals. Blood was collected from the animals and white cells were analyzed for the presence of HTLV-1 cell-associated RNA. If positive, then the animals were sacrificed and DNA isolated from tissues, as described previously (Iordanskiy et al., 2015). Briefly, the animals were sacrificed and various tissues were cut with a fresh razor (0.25–0.75 cm^3^) in PBS. The samples were then treated with a mixture of trypsin/EDTA for 30 min/37°C (10× the volume of tissue chunks). They were then spun using Eppendorf (5415 C) for 5 min at 6,000 rpm. The suspended cell mixture, as well as blood samples, were then used to isolate total cellular DNA using Wizard Genomic DNA Purification Kit (Promega) according to manufacturer’s protocol. All mouse experiments were approved by the George Mason University Institutional Animal Care and Use Committee (IACUC; 0188).

### Statistical Analysis

Standard deviation was calculated in all quantitative experiments done in triplicate. All *p*-values were calculated using 2-tailed Student’s *t*-tests (Microsoft Excel) and were considered statistically significant when *p* < 0.05.

## Data Availability

All datasets generated for this study are included in the manuscript and/or the Supplementary Files.

## Ethics Statement

This study was carried out in accordance with the recommendations of “IACUC# 0188, The Committee on Animal Research.” The protocol was approved by “The Committee on Animal Research” at GMU.

## Author Contributions

DP, CD, SAS, MP, and MC performed all the experiments. DP wrote the manuscript. CD, MP, HB, RM, and FK contributed the writing support of the manuscript. DP and CD created the figures. DP, HD, BL, JJ, LL, RM, and FK performed the overall direction of research.

## Conflict of Interest Statement

BL was employed by company Ceres Nanosciences, Inc. The remaining authors declare that the research was conducted in the absence of any commercial or financial relationships that could be construed as a potential conflict of interest.
