# Supplementary material for: HTLV-1 Extracellular Vesicles Promote Cell-to-Cell Contact
Source: Front Microbiol. 2019 Sep 18;10:2147. doi: 10.3389/fmicb.2019.02147 (PMC6759572; doi:10.3389/fmicb.2019.02147)
Supplement: Supplementary file 1 [file Data_Sheet_1.docx]

Supplementary Material

**Supplementary Figure 1: Cell Viability.** Uninfected recipient (CEM) cell viability at days 0 and 5. Cells were cultured at 1 x 10^6^ cells/mL.

**Supplementary Figure 2: Monocytes treated with HTLV-1 EVs (±IR).**  Uninfected monocytes (U937) were used as recipient cells for EV treatments and subsequent co-culture with HTLV-1 donor cells (10 Gy IR; HUT102 cells) as described previously in **Fig. 7** for CEM and Jurkat cells.
